# Scene Representations Conveyed by Cortical Feedback to Early Visual Cortex Can Be Described by Line Drawings

**DOI:** 10.1523/JNEUROSCI.0852-19.2019

**Published:** 2019-11-20

**Authors:** Andrew T. Morgan, Lucy S. Petro, Lars Muckli

**Affiliations:** Centre for Cognitive Neuroimaging, Institute of Neuroscience and Psychology, University of Glasgow, Glasgow G12 8QB, United Kingdom

**Keywords:** cortical feedback, fMRI, V1, Brain Reading

## Abstract

Human behavior is dependent on the ability of neuronal circuits to predict the outside world. Neuronal circuits in early visual areas make these predictions based on internal models that are delivered via non-feedforward connections. Despite our extensive knowledge of the feedforward sensory features that drive cortical neurons, we have a limited grasp on the structure of the brain's internal models. Progress in neuroscience therefore depends on our ability to replicate the models that the brain creates internally. Here we record human fMRI data while presenting partially occluded visual scenes. Visual occlusion allows us to experimentally control sensory input to subregions of visual cortex while internal models continue to influence activity in these regions. Because the observed activity is dependent on internal models, but not on sensory input, we have the opportunity to map visual features conveyed by the brain's internal models. Our results show that activity related to internal models in early visual cortex are more related to scene-specific features than to categorical or depth features. We further demonstrate that behavioral line drawings provide a good description of internal model structure representing scene-specific features. These findings extend our understanding of internal models, showing that line drawings provide a window into our brains' internal models of vision.

**SIGNIFICANCE STATEMENT** We find that fMRI activity patterns corresponding to occluded visual information in early visual cortex fill in scene-specific features. Line drawings of the missing scene information correlate with our recorded activity patterns, and thus to internal models. Despite our extensive knowledge of the sensory features that drive cortical neurons, we have a limited grasp on the structure of our brains' internal models. These results therefore constitute an advance to the field of neuroscience by extending our knowledge about the models that our brains construct to efficiently represent and predict the world. Moreover, they link a behavioral measure to these internal models, which play an active role in many components of human behavior, including visual predictions, action planning, and decision making.

## Introduction

The visual system's ability to use internal models is of crucial importance for human behavior. This ability allows us to understand environments based on limited visual information, increasing our chances of survival. A Paleolithic hunter, for example, had to recognize predatory threats even when partially occluded by trees. Similar internal models across individuals also provide shared references, facilitating communication and common goals, both of which are signatures of behavior.

Cortical neurons receive sensory signals and contextual predictions of those sensory signals by internal models as separate sources of input. Neurons in early visual cortex are sensitive to sensory stimulation from small portions of stimulus space, defined by classical receptive fields (Hubel and Wiesel, 1959). Because nearby receptive fields and higher-level representations can provide contextual information about incoming sensory input, cortical neurons also receive signals through lateral connections and feedback. We consider these signals predictions of incoming stimuli formulated by internal models of the world (Keller and Mrsic-Flogel, 2018). Such signals cause neurons to amplify and disamplify feedforward signals based on context (Gilbert and Li, 2013; Phillips et al., 2015).

Although the predictions from internal models are important for behavior, they are challenging to study because they require disentanglement from co-occurring sensory input. One strategy is to homogenize sensory input to subsections of cortex using occlusion and measure cortical feedback from neighboring compartments or top-down connections. We have previously used occlusion to show that cortical feedback provides context to early visual cortex (Muckli et al., 2005, 2015; Smith and Muckli, 2010; Muckli and Petro, 2013; Vetter et al., 2014; Revina et al., 2018) and similar results have been found by other laboratories (Sugita, 1999; Williams et al., 2008; Kok and de Lange, 2014). Here, we extend our understanding of features conveyed by this contextual information during visual occlusion.

Previous studies were restricted to a small image sets (e.g., three by Smith and Muckli, 2010). Here we use a larger image set, allowing us to include two superordinate-level scene descriptions: category and depth. These are two candidate features of internal models shown to modulate V1 responses (Walther et al., 2009; Kravitz et al., 2011). Both features can be derived from scene statistics alone (Oliva and Torralba, 2001; Torralba and Oliva, 2002), so they could be present in V1 responses due to feedforward processing alone (not requiring internal models). However, deriving category and depth information from scenes requires integrating information over larger areas of the visual field than expected from direct communication among early cortical neurons. We therefore expect internal models to play a role in the coding of category and depth in early visual cortex. Previous work supports this hypothesis, suggesting the visual system rapidly extracts global features and transmits them through feedback pathways to aid in early processing (Schyns and Oliva, 1994; Bar, 2004; Oliva and Torralba, 2006). We investigated whether feature predictions from internal models carry category and depth information by presenting partially occluded scenes during fMRI and attempting to read out category and depth information from occluded portions of V1 and V2.

Additionally, we acquired behavioral samples of scene-specific internal model predictions by way of sketched line drawings of occluded portions of visual scenes. Line drawings may embody fundamental components of how our visual systems represent the world (Cavanagh, 2005; Sayim and Cavanagh, 2011). If so, we expect drawings to depict internal model predictions of missing scene information. We investigate this possibility by modeling occluded brain activity using line drawings.

Our data reveal that internal models sent to V1 and V2 contain category, but not depth information. We also show that internal model predictions are more scene-specific than categorical, that scene-specific feedback is well described by orientation information contained in line drawings and that the consistency of line drawings across observers predicts the robustness of representations in feedback. We thus demonstrate that line drawings provide a window into the models that the brain creates internally.

## Materials and Methods

### 

#### 

##### Participants.

Twenty-three healthy individuals (*N* = 18 in the main fMRI experiment: 12 female, age = 26.45 ± 5.70, mean ± SD; N = 5 in the second fMRI experiment: 2 female, age = 26.50 ± 5.58) with normal or corrected-to-normal vision gave written informed consent to participate in this study, in accordance with the institutional guidelines of the local ethics committee of the College of Science and Engineering at the University of Glasgow (CSE01127).

##### Stimuli.

Twenty-four real-world scenes from six categories were chosen from a previously compiled dataset (Walther et al., 2009). Images were displayed in grayscale (matched for global luminance) on a rear-projection screen using a projector system (1024 × 768 resolution, 60 Hz refresh rate). Stimuli spanned 19.5 × 14.7° of visual angle and were presented with the lower-right quadrant occluded by a white box (occluded region spanned ≈ 9° × 7°). A centralized fixation checkerboard (9 × 9 pixels) marked the center of the scene images. Stimuli in Experiment 2 were identical to this, with the exception that the occluder was moved to the upper-right quadrant.

##### Experimental design.

Each of the eight experimental runs consisted of 48 scene presentations (we used 24 different scene images, each scene image was displayed twice per run, totaling 16 repetitions of each scene image across the experiment) and one block of retinotopic mapping stimulation. The 48 scene presentations were organized into six scene blocks: beginning with 12 s of baseline, followed by eight scene presentations (each 12 s), and ending with another 12 s of baseline. Stimuli were flashed at a rate of 5 Hz to maximize the signal-to-noise ratio of the BOLD response (Kay et al., 2008). Each sequence was presented in a pseudorandomized order where individual images were not shown twice in a row. To ensure fixation, we instructed participants to respond via a button press to a temporally random fixation color change. So that participants would attend to the scenes, participants were asked to report the category of the scene being presented during the fixation color change using six randomized response buttons.

We used mapping blocks to localize the cortical representation of the occluded region (Muckli et al., 2005). During each mapping block, subjects viewed contrast-reversing checkerboard stimuli (5 Hz) at three visual locations: Target (lower-right quadrant in main experiment, upper-right quadrant in Experiment 2), Surround (of the target), and Control (remaining 3 quadrants). Each mapping block was displayed for 12 s with a 12 s fixation period following, and mapping blocks were randomly inserted between experimental blocks, once per run. We conducted retinotopic mapping (polar angle and eccentricity) runs separately from the main experiment.

##### fMRI acquisition.

fMRI data were collected at the Centre for Cognitive Neuroimaging, University of Glasgow. T1-weighted anatomical and echoplanar (EPI) images were acquired using a research-dedicated 3T Tim Trio MRI system (Siemens) with a 32-channel head coil and integrated parallel imaging techniques (IPAT factor: 2). Functional scanning used EPI sequences to acquire partial brain volumes aligned to maximize coverage of early visual areas (18 slices; voxel size: 3 mm, isotropic; 0.3 mm inter-slice gap; TR = 1000 ms; TE = 30 ms; matrix size = 70×64; FOV = 210×192 mm). Four runs of the experimental task (804 vol), one run of retinotopic mapping [session 1: polar angle (808 vol); session 2: eccentricity (648 vol)], and a high-resolution anatomical scan (3D MPRAGE, voxel size: 1 mm, isotropic) were performed during each of two scanning sessions.

##### fMRI data preprocessing.

Functional data for each run were corrected for slice time and 3D motion, temporally filtered [high-pass filter with Fourier basis set (6 cycles), linearly de-trended], and spatially normalized to Talairach space using Brain Voyager QX 2.8 (Brain Innovation). No spatial smoothing was performed. These functional data were then overlaid onto their respective anatomical data in the form of an inflated surface. Retinotopic mapping runs were used to define early visual areas V1 and V2 using linear cross-correlation of eight polar angle conditions.

**Figure 1.**
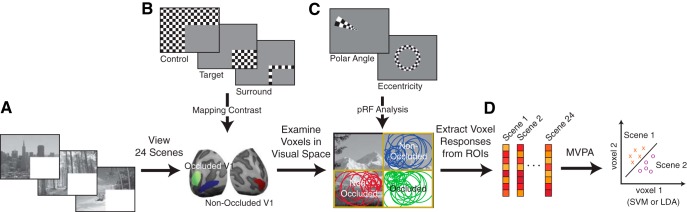
Experimental procedures. ***A***, Participants viewed 24 scenes with their lower right quadrants occluded. Scenes spanned six categories (Beaches, Buildings, Forests, Highways, Industry, and Mountains) and two depths (Near and Far). ***B***, Occluded and non-occluded subsections of early visual cortex were localized using mapping contrasts. ***C***, Retinotopic mapping data were used to separate V1 and V2 and to map pRFs. Voxel pRFs not completely contained by the quadrant of interest (2σ from pRF center) were excluded from further analyses. ***D***, Remaining voxels were included in MVPA.

A general linear model (GLM) with one predictor for each condition (Target > Surround; mapping conditions from experimental runs) was used to define regions-of-interest (ROIs) that responded to the visual target region (lower-right quadrant) and two control regions (upper-right and lower-left quadrants), within V1 and V2. We then performed population receptive field (pRF) analyses (Dumoulin and Wandell, 2008) on all ROI voxels and excluded those voxels whose response profiles were not fully contained (within 2_γ_ of their pRF center) by the respective visual ROI. Last, a conjunction of two GLM contrasts (Target > Surround & Target > Control for Occluded ROIs, and Control > Surround & Control > Target for Non-occluded ROIs) was used to exclude any voxels responding to stimuli presentation outside their respective visual ROI (Fig. 1). Time courses from each selected voxel were then extracted independently per run and a GLM was applied to estimate response amplitudes on a single-block basis. The resulting β weights estimated peak activation for each single block, assuming a standard 2_γ_ hemodynamic response function (HRF).

We modeled activation responses to each scene using a separate regressor time course, also assuming a standard 2_γ_ HRF. For support vector machine (SVM) classification analyses, time courses modeled individual scene image presentations separately, and for representational similarity analysis (RSA), time courses modeled all presentations of a scene contained within a run. This procedure yielded a pattern of voxel activations for each trial or scene, which were then used for proceeding multivoxel pattern analyses (MVPA).

##### Classification analyses.

For SVM classification analyses, voxel activation patterns for each single trial, and parameter estimates (β values) were *z*-scored across voxels. A linear SVM classifier was trained to learn the mapping between a set of all available multivariate observations of brain activity and the particular scene presented, and the classifier was tested on an independent set of test data. Classification analyses were performed using a pairwise multiclass method. Classifier performance was assessed using an *n*-fold leave-one-run-out cross-validation procedure where models were built on [*n* − 1] runs and were tested on the independent *n*th run (repeated for the 8 runs of the experiment). In analyses of category and depth-based classification, individual scene presentation labels were combined based on these distinctions before training and testing of the SVM classifiers. The significance of individual subject testing was assessed using permutation testing of SVM classifiers. We shuffled data labels in training sets and left testing set labels intact, repeating this procedure 1000 times. This procedure resulted in a null classification model around chance-level, and our observed classification value was compared with this distribution to determine the classification significance compared with chance. To determine the group-level distribution of classification performances, we averaged cross-validation folds of individual subject results to arrive at one performance metric for each subject per analysis. We then conducted nonparametric one-sided Wilcoxon signed rank tests to determine whether the distribution of subject performances was above chance level.

Cross-classification analyses were performed similarly to those of our cross-validated classification, but our scene set was split up before model training. Training set sizes consisted of 18 and 22 scenes for category and depth analyses, respectively, and testing sets consisted of the remaining scenes. Because of the large number of possible scene permutations, we conducted 100 iterations of our analyses in each subject. For these analyses, training and testing sets were defined in a pseudorandom manner, where each category or depth was evenly represented within both sets. As in our cross-validated classification, training and testing of models occurred on independent datasets using a leave-one-run-out procedure. Since we performed leave-one-run-out cross-validation on each of the 100 training/testing sets, permutation testing for individual-subject classification significance was not feasible, as it would have required 100,000 tests in each ROI, information type, and subject. We used Wilcoxon rank signed testing to examine individual subject performance. In each of the 100 training/testing sets, we averaged the 8 cross-validated classification performances, resulting in 100 performance values and tested those values against chance-level. To report group-level performance in these analyses, we averaged over cross-validation folds and then averaged performances over the 100 training/testing sets to get an individual subject performance. These values were subjected to one-sided Wilcoxon signed rank tests to assess significance above chance.

To investigate whether activity corresponding to areas near the border of the occluder drives our ability to classify scenes, we systematically reduced the size of the occluded ROI and repeated classification analyses for individual scenes. We shifted the border of the occluded ROI by 0.25° visual angle in each repetition (up to 2.75°). Each successive analysis thus excluded more voxels with pRFs near the border of the occluder. We tested each shifted ROI against chance level (4.17%) at the group level using a Wilcoxon signed rank test with false discovery rate (FDR) correction. We also compared the performance in each reduced-size ROI with the performance of dimensionality-matched control ROIs. Control ROIs had the same number of voxels removed, but we randomly selected voxels rather than being selecting those spatially located near the border of the occluded area. For each individual subject control ROI, random voxel selection occurred 50 times and classifier performances were averaged within subject. We tested each shifted ROI against control ROIs at the group level using FDR-corrected Wilcoxon signed rank tests (paired samples).

##### Line drawings.

Forty-seven individuals consented to participant in a behavioral experiment in which they filled in the occluded subsections of our scene set as line drawings. The experiment consisted of completing scenes using an electronic drawing pen and an Apple iPad Mini tablet (first Generation; screen resolution: 1024 × 768). The pen stroke was six pixels wide and produced purely black lines (no graded pressure settings). The tablet was held at ∼45 cm from the participant to approximate the visual angle of scenes in our fMRI experiments. Participants were given 25 s to complete each drawing with a 5 s break between drawings. The total length of the experiment was 12 min.

Line drawings were averaged over participants to capture the consistency of internal model predictions. Therefore, lines drawn by a large proportion of subjects appear darker than those drawn by a smaller proportion of subjects. Drawings were then scaled between 0 and 1 across the entire scene set, preserving the precision of internal models across scenes. Drawings were then used as input to visual processing models for occluded ROIs.

To measure how well subjects' line drawings captured the occluded portions of the test scenes, we conducted a behavioral experiment in which participants rated how well the line drawings and full scenes matched. Twenty-seven individuals were shown side-by-side comparisons of each scene in its non-occluded form and with its average line drawing in the occluded area. Scenes were presented in random orders and subjects rated the match on a scale from 1 to 7. Subjects were not given any time constraints, and the task took ∼2–3 min. Ratings for the 24 scenes were *z*-scored within each subject and averaged across subjects to obtain a single predictability rating for each occluded scene.

##### Model comparisons.

We compared visual processing models (Weibull, Gist, H-Max, Category, and Depth; for details, see individual model sections) using an RSA framework (Kriegeskorte et al., 2008). In each visual ROI (visible portions of scenes in non-occluded regions and behavioral drawings or actual hidden scenes in occluded regions), representations were calculated for individual channels of each model using a squared Euclidian distance metric [matrix sizes were scene comparisons (276) × model channels].

Model representational dissimilarity matrices (RDMs) were fit to data RDMs using non-negative least-squares (Khaligh-Razavi and Kriegeskorte, 2014) in a cross-validated manner. First, two independent RDMs were calculated using the linear discriminant contrast (LDC) (Walther et al., 2016) method (e.g., Runs 1, 2 vs Runs 3, 4 and Runs 5, 6 vs Runs 7, 8). For each scene comparison, models were fit to the RDM of the 22 other scenes in the first set (e.g., Runs 1, 2 vs 3, 4). This was repeated for all scene comparisons, thus producing a predicted RDM based on model parameters, which was then compared with the second half of the data (e.g., Runs 5, 6 vs 7, 8) using Kendall's Tau-a rank correlation (Nili et al., 2014). This procedure was repeated for all 70 possible split-quarter combinations, and values were averaged over splits to produce one correlation value per subject per ROI/model combination.

Noise ceilings for each cortical area were calculated as the upper and lower bounds on individual subject correlations with the average cortical RDM (Nili et al., 2014). We measured the correlation (Kendall's' Tau-a) of each subject's cortical RDM with the average subject RDM and defined the upper bound of the noise ceiling as the average of these correlation values. We repeated this procedure in a leave-one-subject-out fashion to define the lower bound of the noise ceiling.

These analyses were repeated on subsets of scenes to understand whether there was a relationship between scene predictability and model performance of Gist features computed from line drawings and actual hidden scenes. We ordered the scene set from the most predictable to the least predictable (from our behavioral analysis of scene predictability, using ratings of how well line drawings matched hidden portions of scenes) and performed RSA modeling in 17 bins of 8 scenes in a sliding-window fashion. We tested performance differences between models using Wilcoxon signed rank testing. We controlled the FDR using the Benjamini–Yekutieli procedure (Benjamini et al., 2001) because data are shared between comparisons and this method is capable of controlling FDR while assuming dependence.

##### Weibull model.

The Weibull image contrast model measures the distribution of contrast values for an image and seeks to emulate the X and Y cells in the lateral geniculate (Scholte et al., 2009). It therefore had two outputs (Beta and Gamma statistics, corresponding to X and Y cells), which we calculated within each quadrant in areas extending from fixation to 1.5° and 5° of visual angle, respectively (Groen et al., 2012, 2013).

##### Gist model.

The Gist algorithm measures the distribution of oriented bandpass Gabor filter responses in localized portions of images. Our model used default settings of 16 receptive fields (4 × 4 grid), 8 orientations, and 4 spatial frequencies; (Oliva and Torralba, 2001). This model had a 512-dimensional output.

We performed a split-half reliability analysis on Gist model features computed from line drawings to compare to model performances. We randomly split our subject group in half 50 times (23 subjects in one half and 24 subjects in the other half), averaged their drawings in each iteration (resulting in 2 drawings of each scene), computed Gist features and calculated the correlation between split halves. We calculated the mean and SE of scenes within each bin of eight (organized by scene predictability).

##### H-max model.

The H-Max model is a hierarchical model that gradually combines visual features of higher complexity. Here, we used the output of its fourth sequential stage, C2. The first two stages (S1 and C1) correspond to the simple and complex cells or early visual cortex. Stages S2 and C2 use the same pooling mechanisms as S1 and C1, but pool from the C1 stage and respond most strongly to a particular prototype input pattern. Prototypes were learned from a database of natural images outside of this study (Serre et al., 2007). The output of this model had 2000 dimensions.

##### Category model.

Our Category model had six channels (1 for each scene category). Comparisons between scenes within the same category took distance values of 0 and comparisons between scenes from different categories had distance values of 1.

##### Continuous depth model.

To quantify the depths of our scenes, 10 subjects were asked to assess the depth in meters of each scene in our scene set. To ensure we were probing the perceptual depth of each scene, participants were given minimal guidance on the definition of the term “scene depth”. Depth ratings were converted to a log_10_ scale, bootstrapped via 1000 samples of the mean, and a normal distribution was fit to the resampling histogram for each scene. This produced probability distributions for each scene's depth (Torralba and Oliva, 2002). Squared differences between distribution means were used in our model comparisons analysis. This model had a single dimension as output.

##### Line drawing consistency.

To test whether line drawing consistency across individuals related to robust representations in feedback to V1 and V2, we computed split-half correlations of each scene's average drawing. We randomly split the 47 individual-subject drawings from our behavioral line drawing experiment into two subgroups (23/24 subjects each). We downsampled individual drawings to a resolution of 24 × 32 pixels, averaged within subgroups and correlated (Pearson's *r*) the average drawings from these two subgroups. This procedure was repeated 1000 times, producing 1000 correlation values per scene. The average of the Fisher-transformed correlation values provided a measure of drawing consistency across individuals. We compared drawing consistency to individual scene decodability for each fMRI participant, which was summarized by the average LDC value for each scene (compared with all other scenes) from RSA. These statistics were correlated (Pearson's *r*) in individual subjects across scenes. We evaluated group-level significance using a one-sided Wilcoxon signed rank test.

This measure of line drawing consistency was also summarized within bins of 8 scenes based on scene predictability rankings (see Model comparisons). Within each bin, we included the mean of the 1000 correlation values computed above for each of the eight scenes. These eight values were resampled 1000 times to determine 95% confidence intervals of the mean. We then compared line drawing consistency to the difference between model performance for line drawings and missing scene patches. We entered the mean value for line drawing in each of the 17 bins into a mixed-effects model with random intercept and slope for each of the 18 subjects [Formula in MATLAB fitlme function: Model Performance difference ∼ Line Drawing Consistency + (Line Drawing Consistency | Subject)].

## Results

We blocked feedforward input to subsections of retinotopic visual cortex during an fMRI experiment using a uniform visual occluder that covered one-quarter of the visual field (Smith and Muckli, 2010; Muckli et al., 2015; Revina et al., 2018) while participants viewed 24 real-world scenes. We localized subsections of V1 and V2 that responded either to the occluded portion of the visual field (lower-right image quadrant), or non-occluded visual field (upper-right and lower-left quadrants; Fig. 1). This process yielded three ROIs in each of V1 and V2, hereafter referred to as occluded and non-occluded (either upper-right or lower-left), totaling six ROIs (3 positions in V1, 3 positions in V2). We also mapped pRF locations of individual voxels (Dumoulin and Wandell, 2008) to ensure that their response profiles were within the ROIs in the occluded visual field.

In this experimental design, occluded V1 and V2 neurons receive homogenous white, non-diagnostic feedforward visual input (Fig. 1). Thus, any change in activity pattern is related to non-feedforward input to these areas. This input could be in the form of cortical feedback, where neighboring neurons receive feedforward input, which is sent up the visual hierarchy and subsequently fed back to these occluded portions of V1 and V2. Alternatively, occluded neurons could receive information laterally through horizontal connections. The conservative mapping and large cortical area of occluded ROIs in this study minimizes this second possibility. Thus, activity patterns in occluded V1 and V2 are related to internal model predictions transmitted to the early visual cortex via cortical feedback with only a minimal contribution coming from lateral connections Chemla et al. (2019). To determine whether scene category and depth were related to internal models, we first attempted to decode these two scene characteristics from occluded V1 and V2 responses. Scenes included in this study were therefore balanced across six categories (Beaches, Buildings, Forests, Highways, Industry, and Mountains) and two spatial depths (Near and Far). To explore which scene-specific features are present in internal models, we also compared occluded responses to behavioral predictions of occluded scenes from drawings.

### Decoding high-level scene features from cortical feedback signals to early visual cortex

**Figure 2.**
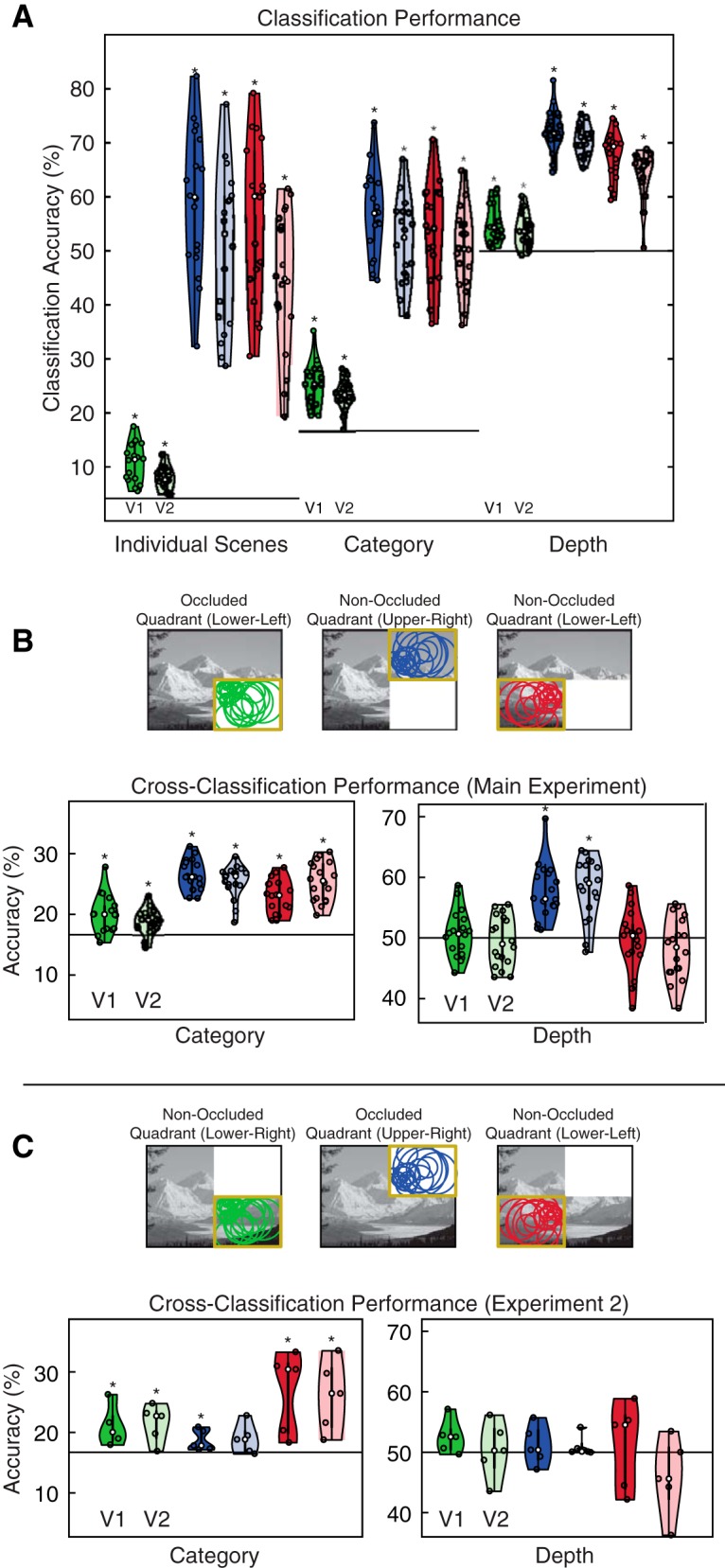
Classification and cross-classification performance. ***A***, Average classifier performances (*N* = 18) are shown for each visual area. Lower-right, upper-right, and lower-left quadrant analyses are shown in green, blue, and red, respectively (the occluder covered the lower-right quadrant in the main experiment and the upper-right quadrant in Experiment 2). Individual subject data are shown as dots and the distribution of these data were computed using kernel density estimation. Asterisks indicate greater than chance-level decoding accuracy (*p* < 0.05, one-sided Wilcoxon signed rank test). Chance level is 4.17% for individual scenes, 16.67% for categories, and 50% for depth (solid black lines in each plot). ***B***, ***C***, Cross-classification performance for Experiments 1 and 2 (*N* = 18 in Experiment 1; *N* = 5 in Experiment 2). Training occurred on 18 and 22 (of 24) randomly chosen scenes in category and depth analyses, respectively, and testing occurred on scenes not used for classifier training. Results were averaged over 100 iterations per subject.

To measure the relationship of Category and Depth with V1 and V2 processing, we used single-trial, linear SVM classification, which has previously been shown to be sensitive in detecting cortical feedback (Smith and Muckli, 2010; Muckli et al., 2015). Initially, we looked at the non-occluded V1 and V2 responses, which contain a mixture of feedforward input, cortical feedback, and lateral interactions. For non-occluded areas, we expected to find the strong decoding of all three types of information in our data, matching what has been found previously (Walther et al., 2009; Smith and Muckli, 2010; Kravitz et al., 2011; Muckli et al., 2015). In our data, we indeed could decode individual scene, category and depth information in these areas (Fig. 2*A*; one-sided Wilcoxon signed rank, all *p* values <0.001).

To address our main question, we asked whether information was decodable from occluded V1 and V2, which receive only cortical feedback and lateral interactions but no direct feedforward input. We were able to decode individual scene, category and depth information in V1 and V2 (Fig. 2*A*), suggesting that internal model predictions in early visual cortex contain these types of information.

Our decoding results were also reliable at the level of individual subjects (Table 1); scene, category, and depth decoding were above chance-level in at least 14 of 18 subjects in nearly all regions tested. We found the weakest decoding for Depth in occluded areas, which were only above chance-level in 10 (occluded V1) and 7 (occluded V2) of 18 subjects.

**Table 1.** Subjects with significant individual (Ind) subject classifications (one-sided Wilcoxon signed rank test)

	V1			V2		
	Ind scenes	Category	Depth	Ind scenes	Category	Depth
Upper-right quadrant (non-occluded)	18	18	18	18	18	18
Lower-left quadrant (non-occluded)	18	18	18	18	18	17
Lower-right quadrant (occluded)	15	17	9	13	15	7

To further test whether occluded V1 and V2 represent higher-level properties of scenes, we performed cross-classification analyses for scene category and depth information. We trained SVM models using responses to a subset of our scenes, leaving out a test set for later cross-classification. For the category analysis, 18 (of 24) scenes were selected, leaving out one scene per category. For depth, we selected 22 (of 24) scenes, leaving out one scene per depth. We tested the classifier on the left-out scenes in a cross-classification approach. Because of the large number of possible image permutations in these analyses, we randomly assigned scenes to training and testing sets 100 times in each subject. Cross-classification of category was successful in occluded and non-occluded areas, although performance was substantially decreased compared with our initial classification. We interpret this decrease in performance as cortical feedback being predominantly related to scene-specific features rather than to category. Cross-classification of depth was only successful in the non-occluded upper-right quadrant, suggesting that depth information is not available in lower visual field responses, regardless of whether feedforward information is available (Fig. 2; Table 2).

**Table 2.** Subjects with significant individual subject cross-classification (Wilcoxon signed rank test)

	V1		V2	
	Category	Depth	Category	Depth
Upper-right quadrant (non-occluded)	18	15	18	15
Lower-left quadrant (non-occluded)	18	5	18	5
Lower-right quadrant (occluded)	15	5	14	6

Our cross-classification results show that responses in both occluded and non-occluded areas of the lower visual field do not contain depth information. This visual field bias limits our ability to assess whether depth information is present in feedback to early visual cortex. We therefore conducted a second fMRI experiment in five subjects using the same scenes, but with the occluder moved to the upper-right quadrant of the visual field. In this experiment, we successfully cross-classified category in V1, with V2 not reaching significance (*p* = 0.091; one-sided Wilcoxon signed rank). Once again, we were not able to cross-classify depth information in the occluded quadrant (Fig. 2). We therefore conclude that internal models of scenes predict category information but not depth information. This finding provides neuroscientific evidence that supports hierarchical views of visual processing. These views suggest that category information is useful for defining global context, which can then be fed back to the early visual cortex to aid with processing, for example in object recognition (Schyns and Oliva, 1994; Fenske et al., 2006; Oliva and Torralba, 2006; Serre et al., 2007).

### Decoding performance in V1 and V2

Differences in occluded and non-occluded V1 and V2 decoding levels are informative for understanding how these areas might make distinct functional contributions to contextual scene features during feedforward and feedback processing. We found decoding to always be higher for feedforward (non-occluded) than for feedback (occluded) conditions in both V1 and V2. Feedforward decoding (non-occluded) was higher in V1 than in V2 in both ROIs (upper and lower ROIs) for individual scenes and category information. For depth decoding, feedforward decoding was higher in V1 than in V2 in the upper-right ROI but not in the lower-left ROI. For cortical feedback, there was no significant difference in the decoding of category or depth between V1 and V2, but there was a significant difference when decoding individual scenes. Since differences in performance can be because of differing numbers of voxels in each ROI, we tested whether the number of voxels in V1 was greater than the number in V2. There was a significant difference in voxel counts for upper-right and lower-left quadrants, but not in the lower-right occluded quadrant (*p* = 0.007, 0.016, and 0.116, respectively, one-sided paired *t* tests). Therefore, the difference between occluded V1 and V2 decoding cannot be explained by ROI size. These findings support previous work suggesting that scene-specific features are more easily read out from V1 than from V2 (Smith and Muckli, 2010). The current study's larger scene set provides additional information, showing that more-general scene features relating to scene category and depth can be read out from occluded V1 and V2 with approximately equal fidelity.

Our occluded V1 and V2 decoding results detect reliable differences in response patterns between scene categories (less so for depth), but the results do not indicate which scene features elicited these patterns. This consideration is particularly important in occluded regions. For instance, if successful decoding of contextual information is confined to voxels with receptive fields near the boundary of the occluder, then it would be difficult to differentiate whether their responses are related to short-range lateral connections or to fMRI signal spill-over. However, if successful decoding is not confined to voxels along the occlusion border, then fMRI signal spillover cannot be a major contributor to our results.

**Figure 3.**
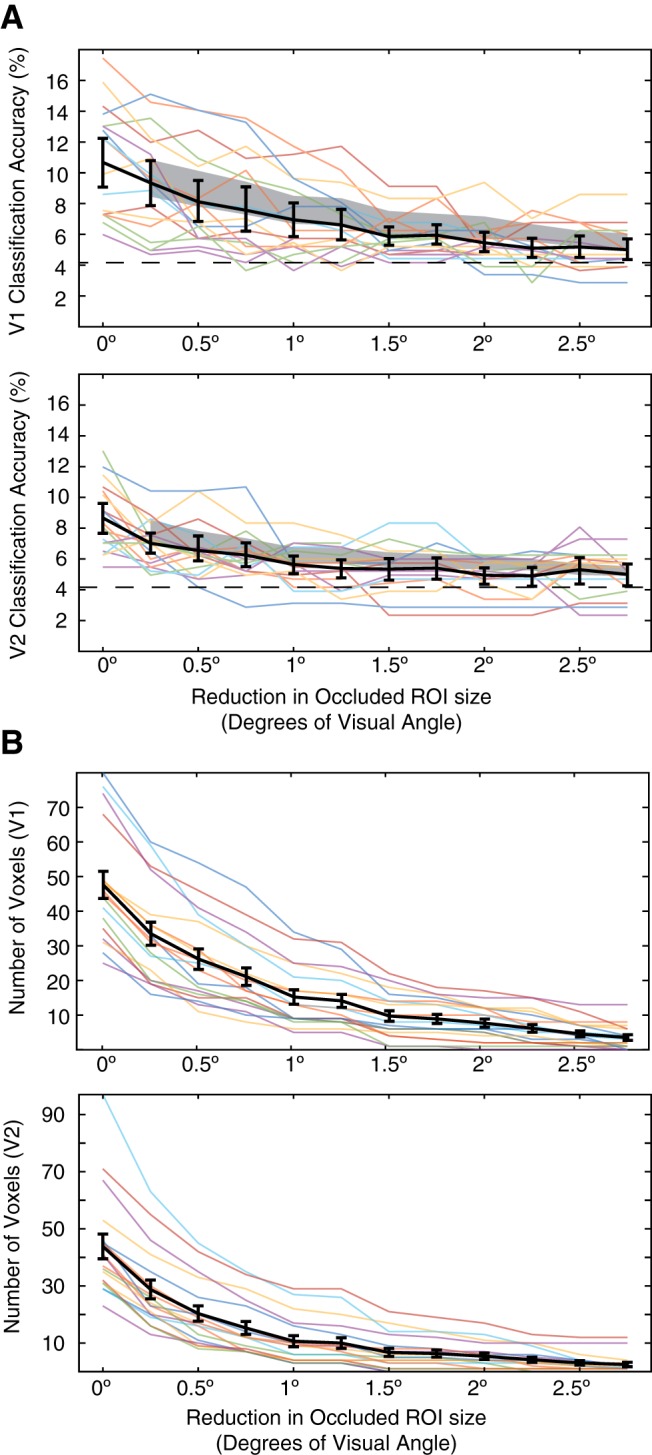
Classification performance in restricted occluded ROIs. ***A***, Classification performance is displayed for V1 and V2 ROIs that were restricted to exclude the area near the border of the occluder. The leftmost column shows classification performance for the full occluded area (as in Fig. 2). Each column to the right shifts the border of the ROI by 0.25° visual angle (up to 2.75°), thus excluding voxels with pRFs near the border of the occluder. Colored lines show individual subject classification performance and black lines shows the mean performance across subjects with 95% CI. The gray shaded area shows performance when the same number of voxels are removed from each ROI, but randomly selected rather than being close to the border of the occluded area (95% CI on the mean). ***B***, The number of voxels included in each subject's ROI are shown by colored lines. Black lines indicate the mean number of voxels (and SE) in each ROI.

To investigate whether activity corresponding to areas near the border of the occluder drives our ability to classify scenes, we systematically reduced the size of the occluded ROI and repeated classification analyses for individual scenes. Figure 3*A* displays the results of 12 such analyses, with the leftmost column showing classification performance for the full occluded area (as in Fig. 2) and each column to the right shifting the border of the ROI by 0.25° visual angle (up to 2.75°). Each successive analysis thus excluded more voxels with pRFs near the border of the occluder.

Importantly, while classification performance decreases as ROIs includes less of the border area, this decrease is not statistically different from a general decrease in ROI size. This is shown by the gray shaded area, which is a dimensionality-matched control analysis where the same number of voxels were removed from each ROI, but were randomly selected rather than being close to the border of the occluded area (95% CI on the mean performance; for the number of voxels in each analysis, see Fig. 3*B*). At the group level, every ROI exhibited above chance-level decoding (4.17%) and was not statistically different from the control analysis (*p* < 0.05, Wilcoxon signed rank). This result shows that the observed decrease in performance is related to the number of voxels included, not to their proximity to the occluder border. It also suggests that the signals we are examining are indeed from cortical feedback and are not because of signal spillover. Instead, these responses could be explained by feedback signals alone, or by interactions between cortical feedback and lateral processing (Erlhagen, 2003; Liang et al., 2017; Chemla et al., 2019).

### Line drawings as internal model read outs

**Figure 4.**
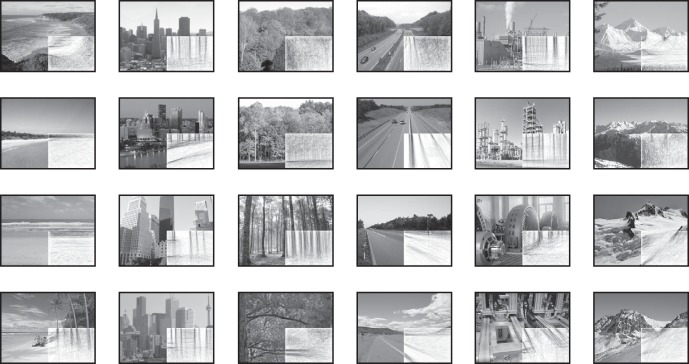
Line drawings are a behavioral measure of internal scene models. Forty-seven individuals filled in the occluded subsections of each scene as a line drawing. Individual subjects' drawings were averaged to create a single group-level drawing for each scene.

We have shown that internal model predictions transmitted to early visual cortex by cortical feedback convey scene category and individual scene information. Next, we wanted to understand whether internal models carry information about predictable occluded features. To derive these features, we conducted a behavioral experiment in which 47 participants drew in the missing scene information that they expected to be behind the white occluder. Figure 4 shows drawings generated by averaging across individual subject's drawings. The coherency of individual drawings is apparent from visual inspection, emphasizing the consistency of internal model predictions across individuals.

**Figure 5.**
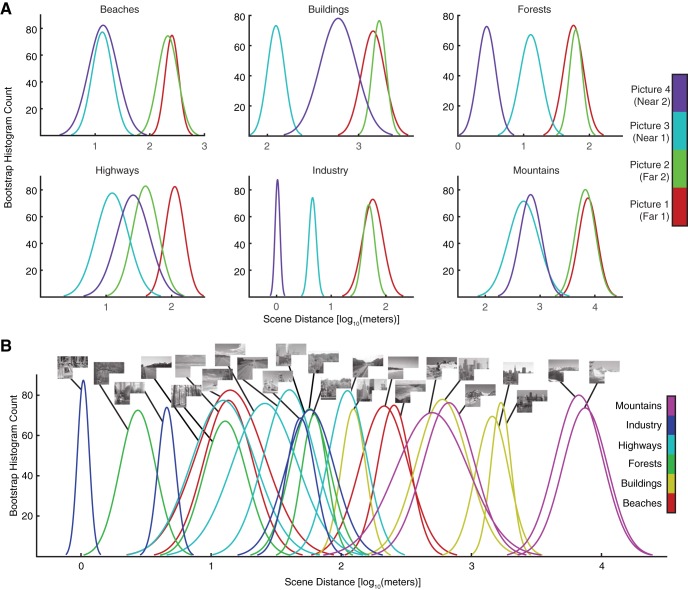
Behavioral depth ratings of scenes. ***A***, Probabilities of depth ratings [log_10_(meters)] for our 24 scenes are shown, grouped by category. Ratings were obtained via a behavioral experiment where subjects were asked to rate the depth of the overall scene in meters (*N* = 10). Individual ratings were converted to log_10_(meters) and the normal distribution of mean ratings for each scene was calculated from a bootstrap histogram (1000 samples). ***B***, Depth ratings for our 24 scenes (identical to ***A*** but plotted together).

**Figure 6.**
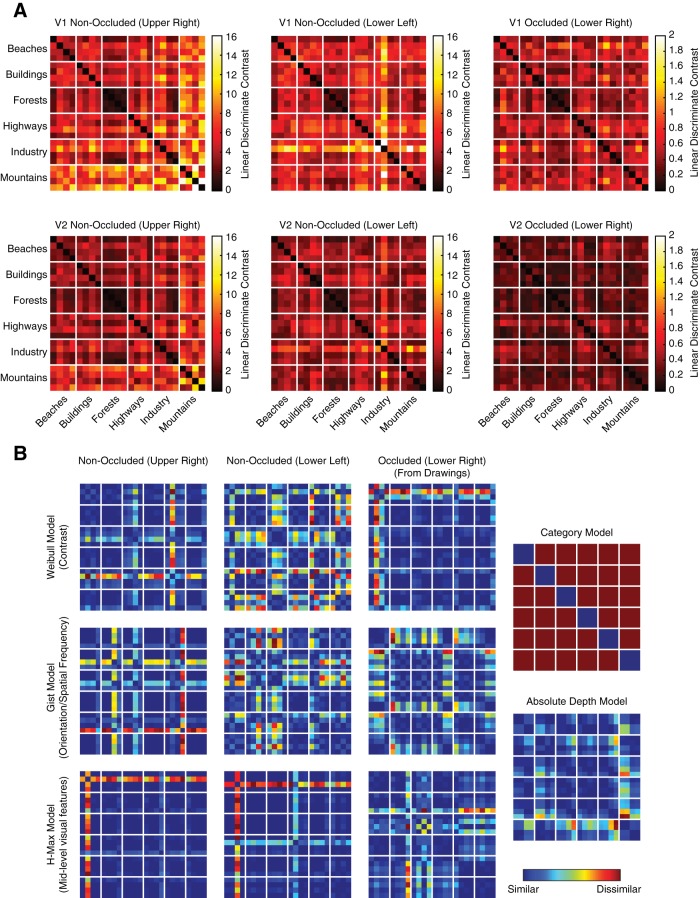
Cortical and model representational dissimilarity matrices. ***A***, Cortical RDMs for all cortical areas tested. ***B***, Model RDMs before fitting to cortical data (shown in arbitrary units).

Using line drawings as behaviorally defined predictions, we used three visual feature models to predict scene representations in V1 and V2: the Weibull model, which corresponds to lateral geniculate contrast processing (Scholte et al., 2009; Groen et al., 2013); the Gist algorithm, which is similar to the orientation and spatial-frequency filters in V1 (Oliva and Torralba, 2001); and the H-Max model (layer C2), which is matched to the tuning properties of intermediate ventral stream areas, such as V4 or posterior IT (Serre et al., 2007). We computed these models on the line drawings and related them to brain activity in occluded areas. To describe brain activity in non-occluded areas, we computed these three models (Weibull, Gist, and H-Max) on the full greyscale scene data (from upper-right and lower-left quadrants). In addition to these scene-specific models, we also included a Category model and a Depth model. The Category model consisted of the six scene categories (Beaches, Buildings, Forests, Highways, Industry, and Mountains). The Depth model was determined by a behavioral experiment in which 10 participants estimated the depth of each scene in meters (Fig. 5). RDMs used to compare models and brain data are shown in Figure 6.

Using RSA (Kriegeskorte et al., 2008), we characterized the multivariate information similarity between scenes in our models and brain data. This allowed us to infer the information content of cortical feedback sent to occluded brain regions. We visualize the RDMs that we used for these comparisons in Figure 6. RDMs for feedforward and feedback brain data from the six ROIs are averaged over subjects and model RDMs are shown before cross-validated fitting to individual-subject brain data. Inspection of brain data RDMs reveals that forest scenes are represented similarly to each other (low dissimilarity measures) in both feedforward and feedback activity patterns. Further, we can already see some similarities between models and brain data. For example, RDMs from the non-occluded upper visual field (Fig. 6*A*, left column) and the absolute depth model (Fig. 6*B*, bottom right) have similar structure. This observation supports what we observed in Figure 2 in non-occluded upper visual field ROIs using SVM classification, which show depth information in upper, but not lower, ROIs.

**Figure 7.**
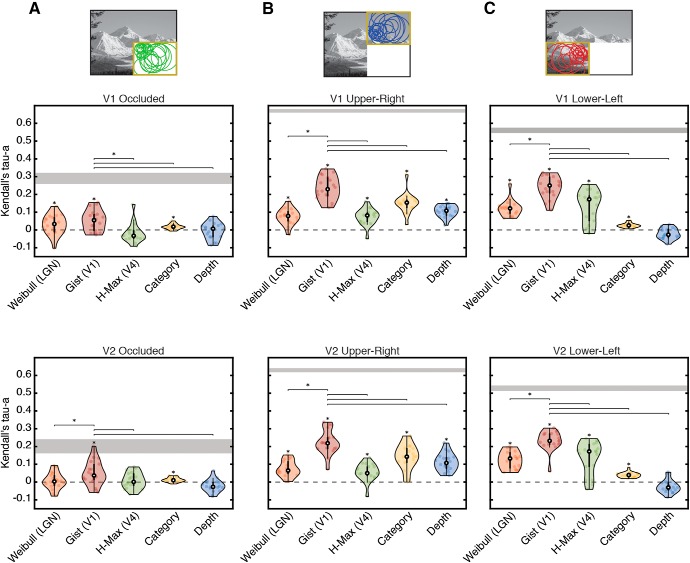
Comparison of scene-specific and global models with cortical representations. The similarity of each model with cortical representations in (***A***) occluded and (***B***, ***C***) non-occluded quadrants of V1 and V2 is shown as a rank correlation (Kendall's Tau-a). The Weibull (LGN contrast processing), Gist (orientation and spatial-frequency processing), and H-Max (mid-level visual feature processing) model features were computed from visible scenes for non-occluded areas, and from line drawings for occluded areas. Global feature models included Category and Depth (depth measurements were determined in a separate behavioral experiment). Individual subject data are shown as dots, and data distributions were computed using kernel density estimation. Asterisks directly above data indicate significantly greater than zero correlations (*p* < 0.05, one-sided Wilcoxon signed rank test). Lines between models indicate significant differences in performance between the Gist model (the best performing model in all areas) and other models. Noise ceilings were calculated as the upper and lower bounds on individual subject correlations with an average representational structure.

Correlations between fitted models and brain data are shown in Figure 7. In occluded V1 (Fig. 7*A*, top), the Weibull, Gist and Category models are all significantly correlated with internal models from cortical feedback (*p* < 0.001, one-sided Wilcoxon signed rank). Interestingly, the orientation information in line drawings (from the Gist model) is significantly more similar to occluded V1 than Category is (*p* = 0.048, two-sided Wilcoxon signed rank). This finding indicates that internal model predictions read out from occluded V1 by cortical feedback are better described by orientation information from line drawings than they are by Category.

In occluded V2 (Fig. 7*A*, bottom), the Gist and Category models are significantly correlated with internal model predictions from feedback. Here again, the Gist model is the highest-correlated model [higher than both Weibull and H-Max models (*p* = 0.039 and 0.025, respectively), but not higher than Category (*p* = 0.071)]. In occluded V2, individual subject correlations of the Gist model with feedback reach into the noise ceiling, indicating that orientation information in line drawings is a good model description for this dataset.

In non-occluded V1 and V2, all models are significantly correlated with brain data other than the Depth model in the lower-left quadrant. The Gist model performs significantly better than any other model (*p* < 0.01) in both non-occluded V1 and V2 (Fig. 7*B*,*C*). This corresponds to the known language of early visual cortex, which is thought to respond to orientation information from feedforward visual input. In this context, our results reveal that cortical feedback to V1 and V2 is also translated into this “orientation language”.

### Line drawing consistency predicts feedback decodability

Our results have shown that early visual cortex responds to visual information hidden from view and that these responses are well described by orientation information from line drawings. Further, many line drawings appear consistent across individuals, which might relate to increased contextual information for informing predictions about occluded scene features. If drawing consistency is related to contextual information availability, we would expect a positive relationship between the consistency of line drawings across individuals and robust representations in feedback to V1 and V2. To test this hypothesis, we computed drawing consistency using split-half correlations of each scene's average drawing (*r* = 0.78 ± 0.091, mean ± SD) and related these measures to individual scene decodability in each subject (the average LDC value for each scene from RSA). Results from this analysis showed that line drawing consistency and scene decodability were indeed correlated at the group-level in both V1 and V2 (*r* = 0.18, *p* = 0.013; *r* = 0.14, *p* = 0.016, respectively; one-sided Wilcoxon signed rank test). These results provide further evidence that line drawings offer insight to predictions made by internal models.

### Line drawings outperform missing scene information as models of feedback

Line drawings can be thought of as simplified representations of visual scenes. They lack many real-world characteristics but capture essential structure required for visual interpretation (Walther et al., 2011). Based on this description, we hypothesized in our previous analyses that line drawings are effective models of cortical activity in occluded scenes because they convey some of the visual information filled in by the brain. Yet this leaves open the possibility that model features derived from actual missing scene patches would be better models of this cortical activity because they contain richer visual information that could also make up predictions by internal models. We therefore wanted to compare models derived from line drawings to models derived from actual missing scene patches. We focused on the Gist model, as it was the best-performing model in describing both feedforward and feedback activity (Fig. 7).

**Figure 8.**
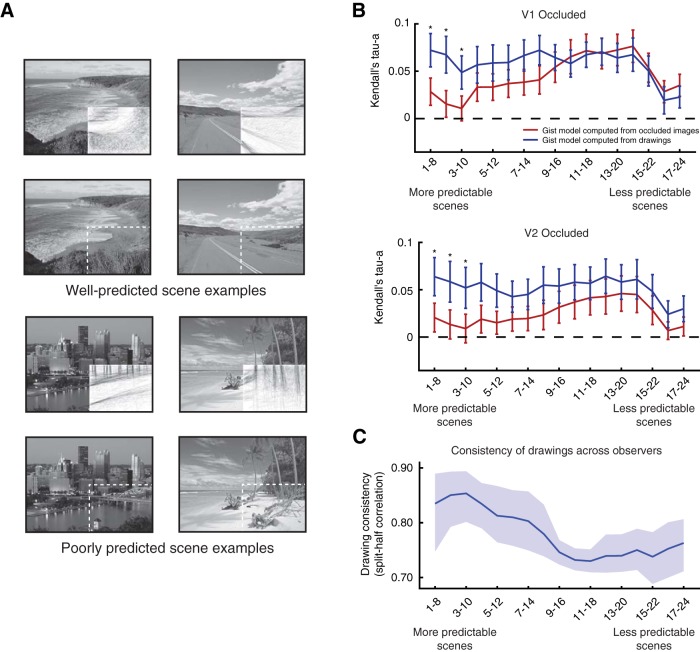
Comparing models of feedback from line drawings to actual missing scene information. ***A***, Examples of well predicted and poorly predicted scenes. ***B***, Occluded V1 and V2 correlations with Gist features computed from line drawings or actual hidden scenes are shown. RSA model comparisons were conducted in bins of eight scenes, which were organized by scene predictability. Mean and SE of correlation values are shown for each model and asterisks indicate significant differences between performances of the two models (*p* < 0.05, Wilcoxon signed rank test). ***C***, The consistency of line drawings across observers is shown in the same bins used in ***B***. The mean and 95% confidence interval for each bin is shown.

Hidden scene patches are not always easily predicted, however, and can have very different structure from that portrayed by line drawings (examples shown in Fig. 8*A*). To account for potential discrepancies in the scene features represented by the two models, we conducted a behavioral experiment in which 27 individuals rated how well (on a scale from 1 to 7) line drawings and full scenes matched in side-by-side comparisons. Ratings can be interpreted as the predictability of the occluded scene features. Based on these ratings, we ordered scenes from most predictable to least predictable and repeated our previously described RSA modeling in bins of eight scenes in a sliding-window fashion. Figure 8*B* shows how well models predict occluded cortical activity patterns. Surprisingly, models derived from hidden scene patches do not outperform line drawings in any of the 17 bins. Furthermore, models built from line drawings of the most predictable scenes significantly outperform models built from hidden scenes patches in both V1 and V2 (*p* < 0.05, two-sided Wilcoxon signed rank test within each bin, FDR corrected to *q* < 0.05), while model performance converges as scenes become less predictable. These results show that models built from line drawings are more similar to cortical predictions of occluded visual information than models built from missing scene patches, at least in predictable scenes, suggesting that predictions in V1 and V2 do not fill in all missing visual information. Instead, feedback only conveys a simplified structure of occluded scene regions.

Does the consistency of line drawings across observers indicate how well the line drawing model explains feedback data? Intersubject consistency of line drawings could be a useful measure of how strongly surround information directs observers toward predicting the same line drawing model. Such a measure would be applicable for studies investigating object occlusion where occluded information is not available (for instance, objects occluding each other in an image database). We computed line drawing consistency across individuals (investigated in the previous section) within each scene bin (Fig. 8*C*). This measure of available contextual information does not require seeing the occluded scene regions and is correlated with our predictability measure (Spearman's ρ = 0.356, *p* < 0.001 between drawing consistency and predictability rankings). The consistency of drawings across individuals also predicted the performance advantage of line drawings over occluded scene patches in V1 (*t* = 2.95, *p* = 0.0034, mixed-effects model with random intercept and slope for each subject), but not in V2 (*t* = 1.63, *p* = 0.10).

Overall, our results corroborate previous work showing that internal models conveyed by cortical feedback contribute to responses of visually occluded brain areas (Williams et al., 2008; Smith and Muckli, 2010; Muckli et al., 2015). Here, we have extended our understanding of cortical feedback by showing that internal model predictions sent to V1 and V2 contain category and scene-specific orientation information. Further, we discovered that responses associated with cortical feedback to V1 and V2 correspond with behavioral predictions of missing visual scene information in the form of line drawings. These results not only show that the earliest stages of cortical sensory processing are informed by behaviorally relevant predictions about the structure of the world, but also demonstrate that line drawings provide a means of accessing the predictions made by the brain's internal models.

## Discussion

Our findings uncovered activation patterns in occluded subregions of early visual cortex informative for determining category and individual scene information about the surrounding images. Contextual feedback to early visual cortex therefore exhibits high-level structure but is more related to scene-specific features. We also found that this scene-specific information correlates with orientation information found in internal model predictions of scenes, which we sampled by asking subjects to complete line drawings of the occluded subsections of scenes. Line drawings have remained largely unchanged during human history and therefore might embody fundamental components of how our visual systems represent the world (Cavanagh, 2005; Sayim and Cavanagh, 2011). Our findings relate this view to that of the visual system as a hierarchical inference network, with V1 acting as a geometric buffer or blackboard (Lee et al., 1998; Lee and Mumford, 2003). In other words, V1 preserves scene information for reference in calculations where spatial precision is required.

Our results support the idea that expected information is present in the early visual cortex without direct visual stimulation (Mumford, 1992; Sugita, 1999; Lee and Mumford, 2003; Williams et al., 2008; Muckli et al., 2015). The prediction of absent visual features can lead to an illusory percept termed *modal completion* (Muckli et al., 2005; Kok and de Lange, 2014; Kok et al., 2016). Modal completion differs from what we have investigated here, as no conscious percept was triggered by the occlusion of visual features. Instead, we anticipate that knowledge captured in internal models supports *amodal completion*, whereby a rudimentary expectation exists about the physical continuation of scene elements into the occluded portion of each scene, without perceptual filling in those features.

How do these relatively small effects in occluded areas influence cortical processing? Optogenetic studies provide evidence for cortical feedback signals influencing neuronal output, and this mechanism is linked to perception (Takahashi et al., 2016). Animal models suggest that cortical layer 5 pyramidal cell spiking is virtually unaffected by stimulation of apical tuft dendrites alone (Larkum et al., 1999; Larkum, 2013), where cortical feedback is largely received. However, neurons are highly sensitive to associative feedback upon receiving feedforward input to their somatic dendrites in a process termed backpropagation-activated Ca^2+^ spike firing (BAC firing; Larkum, 2013), where the coincident arrival of feedforward and feedback tuft inputs leads to bursts of action potentials. Because fMRI BOLD signals are sensitive to energy consumption in both dendritic synaptic processes and spiking activity (Logothetis, 2007, 2008), we might be detecting dendritic stimulation without feedforward input in occluded regions of cortex. These synaptically driven BOLD responses should appear comparatively weaker than those caused by the rigorous BAC firing that occurs when feedforward and feedback inputs are integrated. When combined, these points provide one explanation for how relatively small BOLD changes in occluded regions can be associated with neuronal processes that significantly affect cortical processing before perception (Larkum et al., 2018).

We computed visual feature models from behavioral line drawings and related them to regions receiving cortical feedback signals. One assumption is that the top-down stream takes information from a feature space in a hierarchically higher-level visual area and translates it to a feature space used in a lower-level visual area. This idea of inheriting features of a higher processing stage has been shown in the hierarchically organized macaque face processing network (Schwiedrzik and Freiwald, 2017; Petro and Muckli, 2018). For example, the H-Max model (C2 level) explains a V4- or Posterior IT-like processing stage in a feedforward network (Serre et al., 2007; Kriegeskorte et al., 2008). Using RSA, we found correlations with visual responses in non-occluded areas processing feedforward information. However, H-Max features computed from line drawings did not correlate with cortical feedback (i.e., in occluded area responses). This finding indicates that mid-level features computed by an H-Max-C2 model might describe the feedforward feature space, but that cortical feedback represents different features. Hence, mid-level features summarized by the H-Max model might not contribute essential aspects of internal model predictions transmitted to early visual cortex. In contrast, lower-level visual features of the Gist model correlated with both non-occluded and occluded responses, indicating that this low-level feature space is a common language used by both feedforward and feedback processing. Orientation and spatial frequency (from the Gist model) might be important for predictions in V1 and V2. However, after modeling the behavior tested by line drawings, we conclude that the optimal models for describing cortical feedback responses are those that use fewer features, namely those expressed in line drawings and in the Gist model.

We found that depth information was not present in feedback to V1 and V2. It is important to note that non-occluded V1 and V2 responses in the lower visual field also did not contain depth information, at least for the current stimuli set. This finding highlighted the possibility that our task missed depth information in feedback that was arriving at the upper visual field. We therefore conducted a second fMRI experiment with an upper visual field occluder. This second study replicated the initial finding; depth information was again not present in occluded activity (now in the upper visual field), and the non-occluded lower visual field also did not contain depth information. Future studies would therefore need greater sensitivity to test whether scene depth forms part of cortical feedback information and its contribution to processing biases in V1 and V2 for upper versus lower visual field. Several localized early visual cortical patches respond preferentially to objects nearer to the viewer (Lescroart et al., 2015). The ability to systematically map depth properties onto cortex while manipulating feedforward image information availability would enable researchers to resolve feedforward and feedback signal contributions to cortical response properties. Relatedly, the lack of depth information in our results might be due to our visual system's difficulty in estimating depth from 2D images. Future studies should investigate whether 3D environments convey depth more effectively than 2D scenes and whether this information is integrated into internal models.

We found that line drawings replicate the structure of internal models in early visual cortex. Importantly, drawings and brain data were from different groups of subjects. This suggests line drawings contain generalizable features related to the structure of internal models across individuals but begs the question of whether individual differences exist in this relationship. In future, it will be important to study line drawings from individuals that also have brain recordings like those from the current study to understand if differences in participants' line drawings predict differences in the structure of their internal models.

Line drawings tended to extend edges projecting into occluded areas of scenes (Fig. 4). A previous study has shown that top-down signals are necessary to implement long-range contour integration by modulating lateral connections within V1 (Liang et al., 2017). Our results suggest this mechanism could also be involved in predicting edges and lend support to the hypothesis that our visual systems simulate scene features outside our current field-of-view (Intraub, 2014). The behavioral predictions of missing scene features and associated early visual activity patterns in the current study therefore relate to the need for the visual system to extrapolate available sensory information into its surrounding context.

Our findings advance our understanding of the visual features conveyed by cortical feedback. Mental models expressed at the first visual cortical processing stage are closely related to mental models depicted in line drawings. When faced with a blank canvas, it is conceivable that an artist drawing a visual scene represents this scene using cortical feedback processing until their line drawings converge with the internal model of the visual scene. In the present study, line drawings were consistent across individuals. This is presumably because human visual systems have a comparable mechanism for projecting internal model predictions to the cortical stage for visual information, with this stage acting as an active canvas or blackboard (Lee et al., 1998; Lee and Mumford, 2003).

Accessing the brain's internal models is a key advancement in cognitive neuroscience. We studied the visual system because it is well suited for reading out internal models. However, this is only an example of how internal models contribute across the brain. They also play an active role in visual attention (Lee, 2015), higher cognitive functions such as memory and action planning (Kwon and Knill, 2013), decision-making (Wolpert and Landy, 2012), and mental time travel (Buckner and Carroll, 2007). Internal models are affected in mental disorders including major depression (Messina et al., 2016), schizophrenia (Schmack et al., 2015), and autism spectrum disorder (Van de Cruys et al., 2014; Haker et al., 2016). Previous work has shown differences in the characteristics of line drawings made by schizophrenic patients (Kaneda et al., 2010) and in the ability of autistic children to predict objects from fragmented line drawings (Dehaqani et al., 2016). In the context of our results, these studies could be assessing structural differences of internal models in schizophrenia and autism.

By continuing to expand our brain reading of internal models, we can gain new insights into fundamental neuroscientific questions in health and disease.
